# Virus detections among patients with severe acute respiratory illness, Northern Vietnam

**DOI:** 10.1371/journal.pone.0233117

**Published:** 2020-05-12

**Authors:** Yen H. Le, Khanh C. Nguyen, Kristen K. Coleman, Tham T. Nguyen, Son T. Than, Hai H. Phan, Manh D. Nguyen, Nghia D. Ngu, Dan T. Phan, Phuong V. M. Hoang, Long P. Trieu, Emily S. Bailey, Tyler E. Warkentien, Gregory C. Gray

**Affiliations:** 1 Military Institute of Preventive Medicine, Hanoi, Vietnam; 2 National Institute of Hygiene and Epidemiology, Hanoi, Vietnam; 3 Emerging Infectious Diseases Programme, Duke-National University of Singapore, Singapore; 4 Hai Phong Provincial Preventive Medicine Center, Hai Phong, Vietnam; 5 Division of Infectious Diseases, Global Health Institute, Duke University, Durham, North Carolina, United States of America; 6 Naval Medical Research Center-Asia, Singapore, Singapore; 7 Global Health Center, Duke Kunshan University, Kunshan, China; Defense Threat Reduction Agency, UNITED STATES

## Abstract

Severe acute respiratory illness (SARI) is a major cause of death and morbidity in low- and middle-income countries, however, the etiologic agents are often undetermined due to the lack of molecular diagnostics in hospitals and clinics. To examine evidence for select viral infections among patients with SARI in northern Vietnam, we studied 348 nasopharyngeal samples from military and civilian patients admitted to 4 hospitals in the greater Hanoi area from 2017–2019. Initial screening for human respiratory viral pathogens was performed in Hanoi, Vietnam at the National Institute of Hygiene and Epidemiology (NIHE) or the Military Institute of Preventative Medicine (MIPM), and an aliquot was shipped to Duke-NUS Medical School in Singapore for validation. Patient demographics were recorded and used to epidemiologically describe the infections. Among military and civilian cases of SARI, 184 (52.9%) tested positive for one or more respiratory viruses. Influenza A virus was the most prevalent virus detected (64.7%), followed by influenza B virus (29.3%), enterovirus (3.8%), adenovirus (1.1%), and coronavirus (1.1%). Risk factor analyses demonstrated an increased risk of influenza A virus detection among military hospital patients (adjusted OR, 2.0; 95% CI, 1.2–3.2), and an increased risk of influenza B virus detection among patients enrolled in year 2017 (adjusted OR, 7.9; 95% CI, 2.7–22.9). As influenza A and B viruses were commonly associated with SARI and are treatable, SARI patients entering these hospitals would benefit if the hospitals were able to adapt onsite molecular diagnostics.

## Introduction

Although severe acute respiratory illness (SARI) is a major cause of death and morbidity in low- and middle-income countries, the etiology of SARI is often unconfirmed due to the lack of molecular testing capabilities in under-resourced regions. Hospitals and clinics often rely on broad nationwide influenza surveillance data to describe pneumonia and SARI outbreaks, neglecting onsite molecular diagnosis of influenza and non-influenza respiratory viruses as potential etiologic agents. However, efforts beyond utilizing influenza surveillance to describe pneumonia and SARI in Vietnam have been demonstrated as feasible and efficient by the Vietnam Ministry of Health[1].

In addition to influenza viruses, non-influenza respiratory viruses with zoonotic potential are a serious emerging threat to nations where intermixing of large populations of humans and animals occurs[2]. For example, coronaviruses are prone to cross-species transmission[3] resulting in global outbreaks of human respiratory diseases such as severe acute respiratory syndrome (SARS), Middle East respiratory syndrome (MERS), and most recently, coronavirus disease 2019 (COVID-19)[4]. We also see potential for adenoviruses and enteroviruses to jump species[2, 5], and community-acquired pneumonia treatment guidelines highlight the increasingly recognized presence of viruses as lower respiratory pathogens[6]. Furthermore, our recent study describing pneumonia infections in East Malaysia revealed a high prevalence of influenza A and respiratory syncytial viruses in the region[7], suggesting that under-resourced hospitals and clinics in Southeast Asia could benefit from more robust surveillance of respiratory viruses causing pneumonia and SARI.

In an effort to examine evidence for select viral infections among military and civilian hospital patients with SARI in northern Vietnam, we conducted a prospective study of such patients seen at four hospitals in the Hanoi area. We also sought to epidemiologically describe these infections using patient demographics (gender and age-group). We focused upon detecting high-impact respiratory viruses[8] often associated with human epidemics[2].

## Materials and methods

This study was approved by institutional review boards at the National University of Singapore (NUS IRB Ref. No. B-16-196), the National Institute of Hygiene and Epidemiology (IRB-VN01057) and the Naval Medical Research Center-Asia (NMRC-A) Human Research Protection Office (HRPO.2017.0002).

### Patient sample collection and analysis

Informed written consent was used to gain permission to collect nasopharyngeal (NP) swab specimens and clinical questionnaire data from patients with SARI at four hospitals in Vietnam: Hai Phong General Hospital and Military Hospitals 103, 108 and 354. For minors included in the study, written assent was obtained, as well as written consent from their parents or guardians. Study subjects included any patient admitted to the hospital study site having a respiratory illness with acute onset in the previous 10 days, fever of 38°C or higher, and cough. Patients excluded from the study included those admitted to the hospital more than 48 hours prior to examination by the study physician, persons less than 5 years of age, persons with known HIV/AIDS or active tuberculosis, and persons who underwent a surgical procedure within 14 days of examination by the study physician.

Upon collection, clinical specimens were transported on ice to either the Military Institute of Preventive Medicine (MIPM) or the National Institute of Hygiene and Epidemiology (NIHE), both in Hanoi, Vietnam, and preserved at -80°C until screened for a panel of human respiratory viruses (see molecular methods below). Residual original specimens were again preserved at -80°C and shipped on dry ice to the Duke-NUS Laboratory of One Health Research in Singapore. Laboratory staff at Duke-NUS were blind to the results of the assays at NIHE and MIPM. Results from the two efforts were then compared, and the etiologic agent was confirmed. The few discordant were retested and the results agreed upon.

Blood samples from a portion of SARI patients at Hai Phong General Hospital were cultured in BD BACTEC medium (BD Vietnam Co. Ltd, Ho Chi Minh, Vietnam) and tested for bacteremia using Analytical Profile Index.

#### RNA and DNA extraction, RT-PCR/PCR, and DNA sequencing

RNA or DNA extractions were performed using the QIAmp MinElute Virus Spin Kit (Qiagen, Inc., Valencia, CA). Real-time RT-PCR/PCR was conducted on a BioRad CFx96 C1000 Touch Thermal Cycler Real-Time system at NIHE, MIPM, and Duke-NUS in Singapore. Conventional RT-PCR was conducted at Duke-NUS using SuperScript III One-Step RT-PCR System with Platinum Taq DNA Polymerase.

Following common standard operating procedures and previously published PCR/RT-PCR conditions[9–12], NP swabs were examined for molecular evidence of influenza A and B virus (IAV, IBV)[9], adenovirus[10], enterovirus (EV)[11], and coronavirus (CoV)[12]. Primer and probe sequences for targeted pathogens are recorded in S1 Table. A positive and a no-template control were included in each run. Coronavirus-positive samples were sent to Bio Basic Asia Pacific Pte. Ltd. (Singapore) for Sanger sequencing.

### Statistical methods

Study data were imported into STATA version 15.0 (StataCorp, College Station, TX, USA) for statistical analyses. Pearson’s chi-squared or Fisher’s exact tests were used to examine for differences in viral detections by gender, age group, year of study enrollment, and hospital (military vs. civilian). Logistic regression was used to calculate odds ratios (OR) and 95% confidence intervals (CI) for risk factors associated with prevalent viral detections. To further examine these risk factors, stepwise, unconditional logistic regression using a saturated model and backward elimination (exclusion p > 0.05) was used.

## Results

### Virus detections among SARI cases

A total of 348 SARI patients were enrolled at the four hospitals from January 2017 to January 2019, with 151 (43.4%) of the patients enrolled at one of the three military hospitals (Military Hospital 103, 108 and 354) and 197 (56.6%) enrolled at Hai Phong General Hospital (Table 1). The majority of study patients (62.4%) were female and all study patients were aged 15 years or older. All patients studied at Military Hospitals 103, 108 and 354 were enrolled in 2017, and due to multiple administrative requirements and a large dengue epidemic, study enrollments at Hai Phong General Hospital were not uniformly made over time (S1 Fig).

**Table 1:** The distribution of study subjects with severe acute respiratory illness by age, gender, year of study enrollment, and hospital in the Hanoi area.

Risk factors	Total n = 348 (%)	Hai Phong General Hospital n = 197 (%)	Military Hospitals* n = 151 (%)
Gender			
Male	128 (36.8)	50 (25.0)	78 (51.7)
Female	217 (62.4)	144 (73.0)	73 (48.3)
Unknown†	3 (0.8)	3 (1.5)	--
Age quartiles (Q)			
15–24 yrs (Q1)	77 (22.1)	39 (19.8)	38 (25.2)
25–34 yrs (Q2)	75 (21.6)	54 (27.4)	21 (13.9)
35–56 yrs (Q3)	79 (22.7)	36 (18.3)	43 (28.5)
≥ 57 yrs (Q4)	71 (20.4)	22 (11.2)	49 (32.5)
Unknown§	46 (13.2)	46 (23.4)	--
Year of study enrollment			
2017	210 (60.3)	59 (29.9)	151 (100)
2018	103 (29.6)	103 (52.3)	--
2019	35 (10.1)	35 (17.8)	--

*Military Hospitals 103, 108, and 354

†Gender data not recorded

§Age data not recorded

Among the 348 SARI patients, 184 (52.9%) tested positive for one or more respiratory viruses. Fifty-three of the SARI patients from Hai Phong General Hospital received blood culture tests for bacterial infections, and one of those patients tested positive for *Pseudomonas aeruginosa*. Influenza A virus was the most prevalent virus detected (64.7%), followed by influenza B virus (29.3%), enterovirus (3.8%), adenovirus (1.1%), and coronavirus (1.1%), (Table 2). Among the military hospital patients, 103 (68.2%) tested positive for one or more respiratory viruses, and 81 (41.1%) of the civilian hospital patients tested positive for one or more respiratory viruses. The most prevalent viruses identified among military hospital patients were influenza A virus (63.1%) and influenza B virus (31.1%), followed by enterovirus (2.9%), adenovirus (1.9%) and coronavirus 229E (1.0%). The most prevalent viruses identified among civilian hospital patients were influenza A virus (66.7%) and influenza B virus (27.2%), followed by enterovirus (4.9%) and coronavirus HKU1 (1.2%). No civilian hospital patient specimens were confirmed positive for adenovirus.

**Table 2:** Viral detections* among 348 patients with severe acute respiratory illness in the Hanoi area, 2017–2019.

Risk factors	Total n = 348 (%)	IAV+ n = 119 (%)	IBV+ n = 54 (%)	EV+ n = 7 (%)	HAdV+ n = 2 (%)	CoV+ n = 2 (%)
Gender						
Male	128 (36.8)	44 (37.0)	22 (40.7)	2 (28.6)	--	--
Female	217 (62.4)	73 (61.3)	32 (59.3)	5 (71.4)	2 (100)	2 (100)
Unknown†	3 (0.8)	2 (1.7)	--	--	--	--
Age quartiles (Q)						
15–24 yrs (Q1)	77 (22.1)	29 (24.4)	8 (14.8)	3 (42.9)	1 (50.0)	--
25–34 yrs (Q2)	75 (21.6)	29 (24.4)	12 (22.2)	3 (42.9)	1 (50.0)	2 (100)
35–56 yrs (Q3)	79 (22.7)	25 (21.0)	14 (26.0)	1 (14.2)	--	--
≥ 57 yrs (Q4)	71 (20.4)	27 (22.7)	12 (22.2)	--	--	--
Unknown‡	46 (13.2)	9 (7.5)	8 (14.8)	--	--	--
Year of study enrollment						
2017	210 (60.3)	79 (66.4)	49 (90.7)	5 (71.4)	2 (100)	1 (50.0)
2018	103 (29.6)	35 (29.4)	5 (9.3)	2 (28.6)	--	1 (50.0)
2019§	35 (10.1)	5 (4.2)	--	--	--	--
Hospital type						
Military	151 (43.4)	65 (54.6)	32 (59.3)	3 (42.9)	2 (100)	1 (50.0)
Civilian	197 (56.6)	54 (45.4)	22 (40.7)	4 (57.1)	--	1 (50.0)

Abbreviations: IAV, influenza A virus; IBV, influenza B virus; EV, enterovirus; CoV, coronavirus; HAdV, human adenovirus

*Virus detection by RT-PCR/PCR with Ct value ≤ 38

†Gender data not recorded

‡Age data not recorded

§January 2019 only

Age-group and gender were not predictors for specific viral detections. However, Pearson’s chi-squared and Fisher’s exact tests demonstrated statistically significant differences in influenza A and B virus by year of study enrollment and between hospitals (military vs. civilian). Further risk factor analyses demonstrated an increased risk of influenza A virus detection among military hospital patients (adjusted OR, 2.0; 95% CI, 1.2–3.2; Table 3), and an increased risk of influenza B virus detection among patients enrolled in year 2017 (adjusted OR, 7.9; 95% CI, 2.7–22.9; Table 4).

**Table 3:** Unadjusted and adjusted risk factor associations for Influenza A Virus (IAV) detection* among patients with severe acute respiratory illness in the Hanoi area, 2017–2019.

Risk factors	Total No. (%)	IAV+ n = 119 (%)	Unadjusted OR (95% CI)†	Adjusted‡ OR (95% CI)
Gender§				
Male	128 (36.8)	44 (37.6)	1.0 (0.6–1.7)	--
Female	217 (62.4)	73 (62.4)	Reference	--
Age quartiles (Q)‖				
15–24 yrs (Q1)	77 (25.5)	29 (26.4)	Reference	--
25–34 yrs (Q2)	75 (24.8)	29 (26.4)	1.0 (0.5–2.1)	--
35–56 yrs (Q3)	79 (26.2)	25 (22.7)	0.8 (0.4–1.6)	--
≥ 57 yrs (Q4)	71 (23.5)	27 (24.5)	1.0 (0.5–2.1)	--
Year of study enrollment				
2017	210 (60.3)	79 (66.4)	1.2 (0.7–2.0)	--
2018	103 (29.6)	35 (29.4)	Reference	--
2019	35 (10.1)	5 (4.2)	0.3 (0.1–1.0)	--
Hospital type				
Military	151 (43.4)	65 (54.6)	2.0 (1.2–3.2)	2.0 (1.2–3.2)
Civilian	197 (56.6)	54 (45.4)	Reference	Reference

*Influenza A virus detection by real-time RT-PCR with Ct value ≤ 38

†Exact confidence intervals

‡After stepwise, unconditional logistic regression using a saturated model and backward elimination of covariates with p > 0.05

§Gender data missing for some patients (n = 3) removed from calculations

‖Age data missing for some patients (n = 46) removed from calculations

**Table 4:** Unadjusted and adjusted risk factor associations for Influenza B Virus (IBV) detection* among patients with severe acute respiratory illness in the Hanoi area, 2017–2019.

Risk factors	Total No. (%)	IBV+ n = 54 (%)	Unadjusted OR (95% CI)†	Adjusted‡ OR (95% CI)
Gender§				
Male	128 (36.8)	22 (40.7)	1.2 (0.6–2.3)	--
Female	217 (62.4)	32 (59.3)	Reference	--
Age quartiles (Q)‖				
15-24yrs (Q1)	77 (25.5)	8 (17.4)	Reference	--
25-34yrs (Q2)	75 (24.8)	12 (26.1)	1.6 (0.6–4.9)	--
35-56yrs (Q3)	79 (26.2)	14 (30.4)	1.9 (0.7–5.4)	--
≥ 57yrs (Q4)	71 (23.5)	12 (26.1)	1.8 (0.6–5.3)	--
Year of study enrollment				
2017	210 (60.3)	49 (90.7)	6.0 (2.3–19.8)	7.9 (2.7–22.9)
2018	103 (29.6)	5 (9.3)	Reference	Reference
2019	35 (10.1)	--	--	--
Hospital type				
Military	151 (43.4)	32 (59.3)	2.1 (1.1–4.1)	--
Civilian	197 (56.6)	22 (40.7)	Reference	--

*Influenza B virus detection by real-time RT-PCR with Ct value ≤ 38

†Exact confidence intervals

‡After stepwise, unconditional logistic regression using a saturated model and backward elimination of covariates with p > 0.05

§Gender data missing for some patients (n = 3) removed from calculations

‖Age data missing for some patients (n = 46) removed from calculations

**Fig 1:**
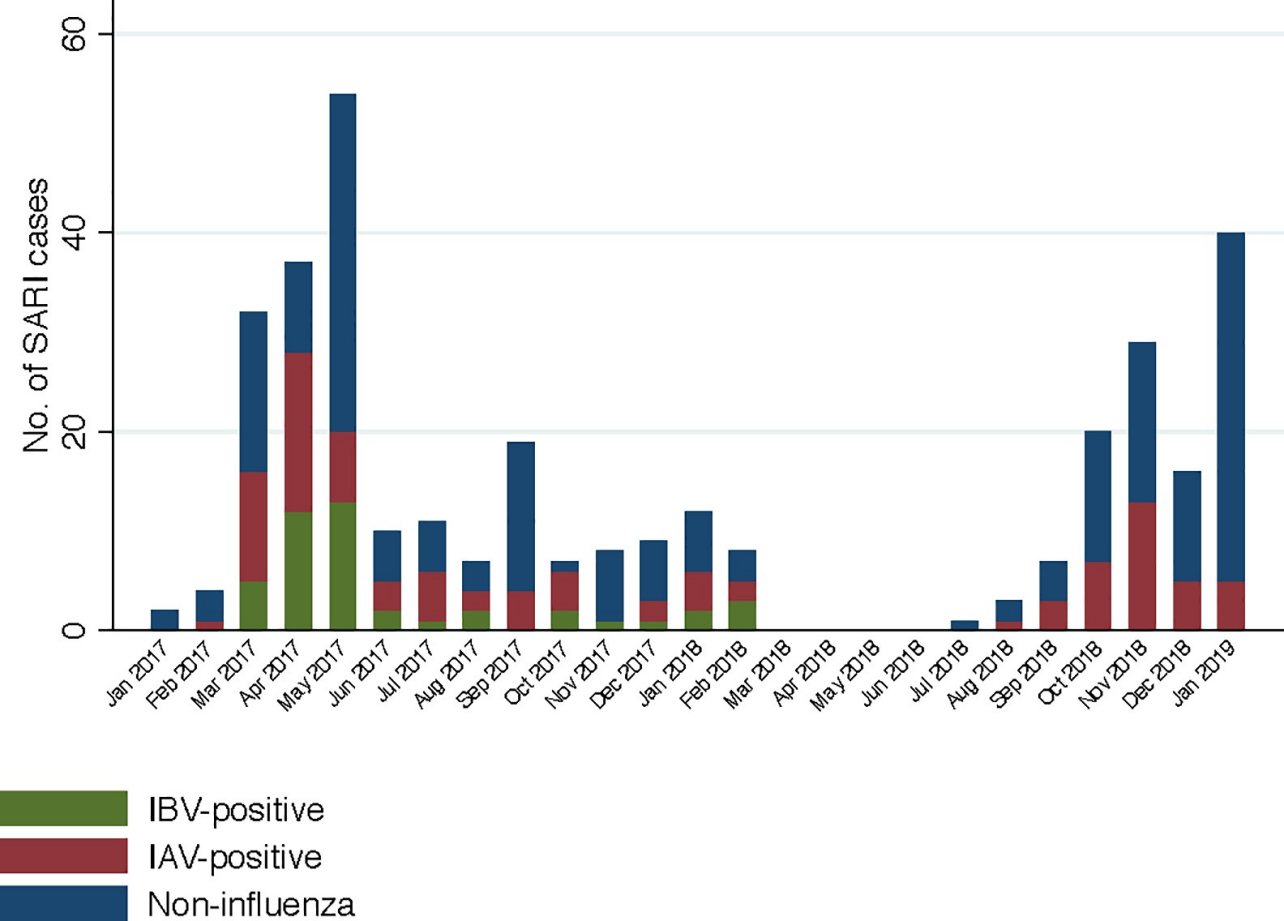
Influenza virus-positive detections among study subjects with Severe Acute Respiratory Illness (SARI) enrolled over time in the Hanoi area, 2017–2019. IBV = influenza B virus; IAV = influenza A virus. Military hospital enrollments ended in December 2017. Hai Phong General Hospital enrollments paused from November 2017 through December 2017 and again from March 2018 to July 2018.

The first case of influenza A virus in this study was detected from a military hospital patient enrolled in February 2017, followed by detections throughout the study, with the highest number of detections in April 2017 and November 2018 (Fig 1). The first case of influenza B virus in this study was detected from a civilian hospital patient in March 2017, followed by detections throughout the study until February 2018, with the highest number of detections in April and May 2017. Influenza B virus was not detected from March 2018 through January 2019.

There were no confirmed cases of co-infection with influenza A and B viruses in our study. Cases of influenza A and B virus were found among all study age quartiles and genders (Table 2). There was one case of co-infection with enterovirus and coronavirus 229E including a female military hospital patient 28 years of age. Enterovirus cases were found among all genders and age quartiles except for patients ≥ 57 years of age. The 2 coronavirus cases were found among female patients aged 25–34 years. The 2 adenovirus cases were found among female military hospital patients aged 15–34 years.

## Discussion

Viruses identified in our study are known to sometimes cause severe respiratory disease in humans. Similar to pneumonia etiology studies in Vietnam and central China[1, 13, 14], influenza A virus was the most common virus detected among patients in our study. Notably, influenza B virus was not detected during the final 10 months of the study (March 2018 through January 2019), which reflects the near absence of influenza B virus cases reported in Vietnam during that time[15]. However, our study enrollments paused from March 2018 to July 2018 due to administrative requirements and therefore the number of influenza cases at our hospital study sites during that time is unknown. The increased risk of influenza A virus infection among military hospital patients in our study was expected, as military trainees often seek care at these hospitals and rates of respiratory illness among military trainees tends to exceed those found in civilian populations[16].

Enterovirus is a widespread pathogen among children and is often overlooked in adults[17, 18]. In our study, 6 of the 7 enterovirus infections were among young adults, which could have been attributable to their probable contact with young children. Additionally, 2 pairs of the enterovirus infections occurred within 1–2 weeks, suggesting that these could have been clusters. Lastly, 5 of the enterovirus infections were detected from late March to June 2017, which reflects seasonal patterns of epidemics of enterovirus 71 in some Asian countries[19]. Our study was limited in that we did not subtype the enterovirus-positive patient samples. It is also possible that the enterovirus assays used in our study could have detected rhinoviruses, as cross-reactivity between rhinoviruses and enteroviruses has previously been demonstrated[20].

Adenovirus is an important pathogen among military and civilian populations[21, 22]. Adenovirus infections among civilian populations are increasingly reported in Southeast Asia[23–26], therefore it is interesting that no adenovirus cases were confirmed among the civilian hospital patients enrolled in our study. This observation suggests that adenovirus might be largely restricted to the military population and military hospitals in northern Vietnam. However, it is important to note that adenovirus infections are often found among pediatric populations, and patients < 15 years of age were not enrolled in our study. Although only 2 cases of adenovirus infection were confirmed in our study, our approach was limited in that we did not subtype the adenovirus-positive samples, which has been demonstrated as a useful tool in identifying emerging and virulent adenoviruses among populations[26, 27].

In contrast to the enterovirus- and adenovirus-positive samples in our study, we subtyped the coronavirus-positive samples. Coronavirus HKU1 and 229E were identified, which are both common causes of mild respiratory illness. However, both study patients with coronavirus were admitted to the intensive care unit (ICU) with symptoms of respiratory failure requiring oxygen therapy. The study patient with coronavirus 229E had a confirmed co-infection with enterovirus. We suspect that the severity of respiratory illness among the 2 young adult patients infected with coronavirus could be due to co-infection, although the effect of viral co-infections on disease severity are unclear for some common respiratory viruses[28] and the study patient with coronavirus HKU1 did not have a confirmed co-infection. However, we screened patients only for the respiratory viruses mentioned in our study, and therefore we could not rule out co-infections with other viral or non-viral pathogens.

Microbiological testing for bacterial infections among SARI and pneumonia patients is not routine procedure at the study hospitals and therefore only 53 of the SARI study patients received blood culture tests, and only one of those patients tested positive for *P*. *aeruginosa* bacteremia. This patient was a 30-year-old female from the civilian hospital who also tested positive for influenza A virus. Our study was limited in that we also did not have the capacity to test patient samples for human parainfluenza, human metapneumovirus, or RSV. However, these diseases are primarily found in patients outside of this study’s recruited age range, such as infants, young children, and older adults. Nevertheless, human parainfluenza viruses are members of high-impact viral groups with pandemic potential[8], and surveilling for these viruses among SARI and pneumonia patients in our study’s age range could be valuable.

With an overall goal to reduce SARI in Vietnam, this study aimed to examine evidence for select viral infections among SARI patients in northern Vietnam. We chose to focus on high-impact respiratory viruses[8] that are often associated with human epidemics[2]. As influenza viruses were commonly associated with SARI and are treatable, the hospitals in this study could benefit from onsite molecular diagnostic capability. Additionally, our results display enterovirus, adenovirus and coronavirus infections among the SARI cases, suggesting that cities in northern Vietnam could benefit also from local surveillance of non-influenza respiratory viruses.

## Supporting information

S1 Fig Study subjects with Severe Acute Respiratory Illness (SARI) enrolled over time, hai phong general hospital vs. three military hospitals in the Hanoi area, 2017–2019. Military hospital enrollments ended in December 2017. Hai Phong General Hospital enrollments paused from November 2017 through December 2017 and again from March 2018 to July 2018.
(TIF)

S1 Table Primer and probe sequences for RT-PCR/PCR detection* of targeted viruses among patients with severe acute respiratory illness in the Hanoi area, 2017–2019. (DOC)
