# Supplementary figures and images for: Virus detections among patients with severe acute respiratory illness, Northern Vietnam
Source: PLoS One. 2020 May 12;15(5):e0233117. doi: 10.1371/journal.pone.0233117 (PMC7217455; doi:10.1371/journal.pone.0233117)

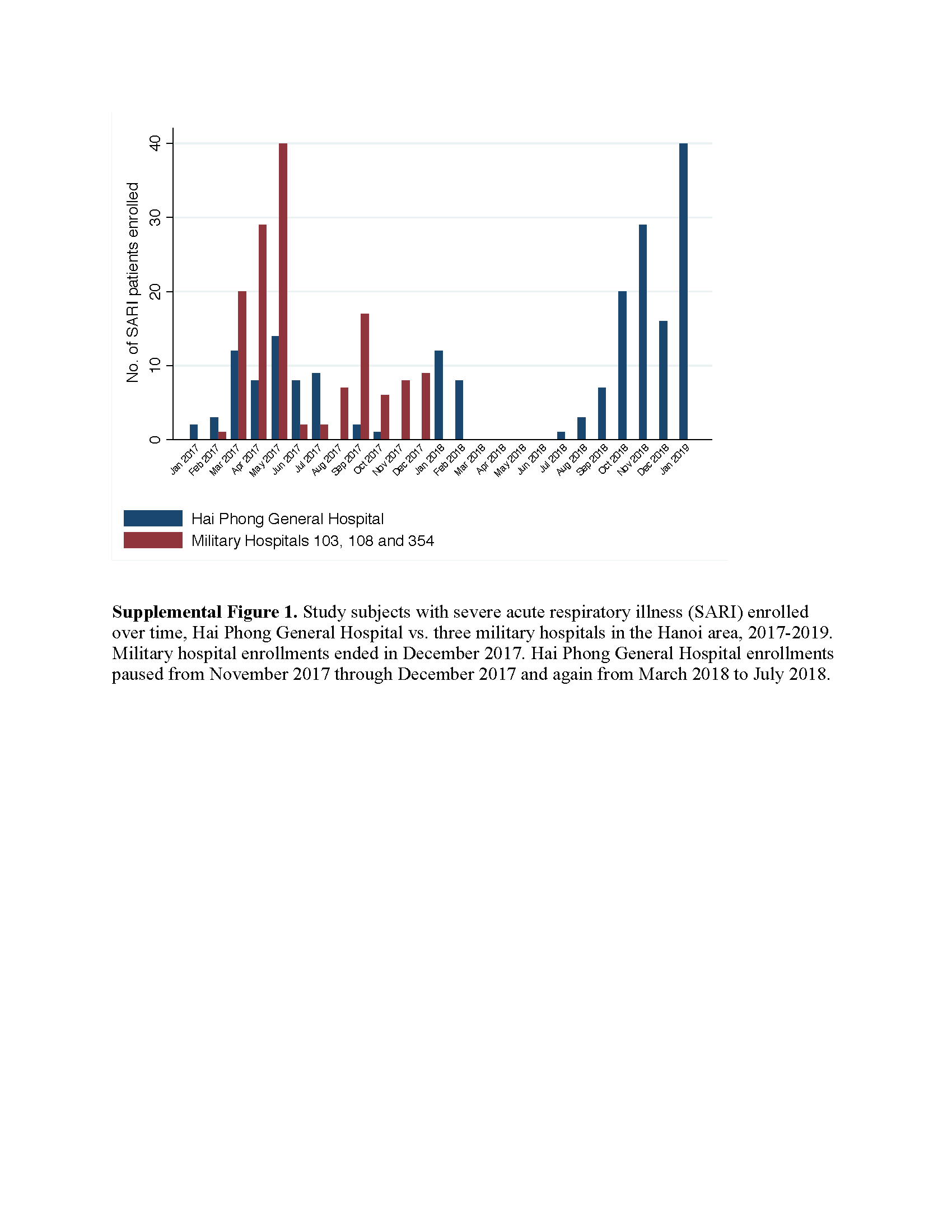

Supplement: S1 Fig — Military hospital enrollments ended in December 2017. Hai Phong General Hospital enrollments paused from November 2017 through December 2017 and again from March 2018 to July 2018. (TIF) [file pone.0233117.s001.tif]
