# Supplementary material for: Virus detections among patients with severe acute respiratory illness, Northern Vietnam
Source: PLoS One. 2020 May 12;15(5):e0233117. doi: 10.1371/journal.pone.0233117 (PMC7217455; doi:10.1371/journal.pone.0233117)
Supplement: S1 Table — (DOC) [file pone.0233117.s002.doc]

| Supplemental Table 1: Primer and probe sequences for RT-PCR/PCR detection* of targeted viruses among patients with severe acute respiratory illness in the Hanoi area, 2017-2019 | | | |  |  |
| --- | --- | --- | --- | --- | --- |
|  | **Forward primer(s)** | **Reverse primer(s)** | **Probe(s)** | **Gene or Target** | **Reference** |
| **IAV** | 5’-GAC-CRA-TCC-TGT-CAC-CTC-TGA-C-3’ | 5’-AGG-GCA-TTY-TGG-ACA-AAK-CGT-CTA-3’ | 5’-FAM-TGC-AGT-CCT-CGC-TCA-CTG-GGC-ACG-BHQ 1-3’ | Matrix | Centers for Disease Control and Prevention, 2017 [9] |
| **IBV** | 5’-TCC-TCA-AYT-CAC-TCT-TCG-AGC-G-3’ | 5’-CGG-TGC-TCT-TGA-CCA-AAT-TGG-3’ | 5’-FAM-CCA-ATT-CGA-GCA-GCT-GAA-ACT-GCG-GTG-BHQ 1-3’ | Matrix | Centers for Disease Control and Prevention, 2017 [9] |
| **CoV** | 5'-GGTTGGGACTATCCTAAGTGTGA-3' | 5'-CCATCATCAGATAGAATCATCATA-3' | -- | *RdRp* | Lelli *et al*, 2013 [12] |
| **EV** | 5’-GGCCCCTGAATGCGGCTAATCC-3’ | 5’-GCGATTGTCACCATWAGCAGYCA-3’ | 5’-FAM-CCGACTACTTTGGGWGTCCGTGT-IBFQ-3’ | 5’NTR | Oberste *et al.*, 2012 [11] |
| **ADV** | 5’-CAG-GAC-GCY-TCG-GAG-TAC-CTG-A-3’ | 5’-CGG-TGG-TCA-CAT-CGT-GGG-T-3’  5’-GCT-GAA-GTA-CGT-VTC-GGT-GGC-3’  5’-GGT-GAA-GTA-GGT-GTC-CGT-GGC-3’ | 5’-FAM-TGG-TGC-AGT-TYG-CCC-G-MGB(NFQ)-3’ | Hexon | Bil-Lula *et al.*, 2012 [10] |
| Abbreviations: adenovirus (ADV); coronavirus (CoV); enterovirus (EV); influenza A virus (IAV); influenza B virus (IBV)  *RT-PCR/PCR assays were performed separately under their previously published conditions [9-12] | | | | | |
